# Exploring the Association between Neighborhood Blue Space and Self-Rated Health among Elderly Adults: Evidence from Guangzhou, China

**DOI:** 10.3390/ijerph192316342

**Published:** 2022-12-06

**Authors:** Yujie Chen, Yuan Yuan, Yuquan Zhou

**Affiliations:** 1 School of Geographic and Biologic Information, Nanjing University of Posts and Telecommunications, Nanjing 210023, China; 2 School of Geography and Planning, Sun Yat-sen University, Guangzhou 510006, China; 3 Guangdong Key Laboratory for Urbanization and Geo-Simulation, Guangzhou 510006, China; 4 Department of Urban Planning and Spatial Analysis, Sol Price School of Public Policy, University of Southern California, Los Angeles, CA 90089, USA

**Keywords:** healthy community, blue space, self-rated health, elderly adults, neighborhood effect

## Abstract

Blue spaces is associated with self-rated health (SRH), but little is known about the pathways underlying this association among Chinese urban elderly individuals. Based on neighborhood effect theory, this study examined the relationship between neighborhood blue spaces and SRH among elderly individuals using data from a questionnaire survey conducted in Guangzhou, remote sensing images, street views, and environmental information in the context of a Chinese megacity. In addition, multilevel linear model and mediating effect model empirical analyses were performed. Results showed that first, the SRH of the elderly was associated with individual- and neighborhood-level factors. Second, the multilevel mediation model revealed that multiple biopsychosocial pathways existed between neighborhood blue spaces and the SRH of the elderly, specifically, the blue space characteristics related to the SRH of the elderly via the mediating effect of stress. Third, owing to demographic characteristics and socioeconomic status, the stratified analyses also indicated a strong association between neighborhood blue spaces and SRH outcomes in the older and low-income groups. The mediating effect of stress in the age and income groups was also observed, and the mediation pathways and group differences were confirmed in the context of Chinese cities. This research enriches the empirical literature on blue spaces and elderly health from a multidisciplinary perspective and suggests the need for “healthy neighborhood” and “health-aging” planning in Chinese settings.

## 1. Introduction

Population aging and urbanization are two prominent characteristics that comprise the major force shaping the 21st century, especially in developing countries. According to the World Health Organization, the proportion of the world’s population over the age of 60 years will reach 22% in 2050, and 80% of the elderly population will be living in low- and middle-income countries [1]. Improving the elderly’s health in urban areas has become an important issue. As an important space carrier of elderly life, healthy communities play a key role in enabling such individuals to live long and healthy lives while fostering fair and sustainable societies.

The United Nations Population Division reported that 9.318% of the total population of China is aged 65 years or above. Experiencing rapid population aging, China is home to the largest number of elderly adults globally. Rapid urbanization considerably decreased access to natural outdoor environments and posed health challenges for elderly adults. In many countries, “healthy aging” has become a strategy for coping with population aging. Scholars, practitioners, and policymakers also increased their attention to the benefits of the natural environment for the health of elderly individuals. In the context of “aging-in-place”, health outcomes result from interactions among the elderly, therapeutic environment landscapes, and the broad social context that shapes such settings [2]. As the neighborhood is the space carrier of elderly daily life, the neighborhood effect can influence the health, social outcomes, and behaviors of the elderly in the short or long term [3]. As a therapeutic landscape, neighborhood blue spaces provide spaces and places to the elderly to rest, seek entertainment, exercise, and engage in social communication.

Within the urban setting, blue spaces, also known as “aquatic environments”, refer to all visible surface waters in outdoor environments, including lakes, rivers, and coastal waters [4]. According to the definition of the Bluehealth project funded by the European Commission, a blue space is an outdoor environment, either natural or constructed, which prominently features water and is accessible to humans either proximally (being in, on, or near water) or distally/virtually (being able to see, hear, or sense water) [5]. The therapeutic connection between people and water (blue spaces) is widely acknowledged [6,7]. For the elderly, epidemiological evidence showed a significant positive relationship between blue space and health outcomes, including their general health [8], mental health [9], and subjective wellbeing [10].

Blue spaces play a crucial role in people’s health through health-related appropriations, including use (restoration and increased physical activity), experience (environmental experiences such as getting fresh air), social interactions (interaction with other individuals), and meaning (aesthetic pleasure) [11].

Previous studies indicated several potential biopsychosocial pathways linking blue spaces to health [12,13]. (1) Mitigation (e.g., reducing environmental harm of urban heat island effect, air pollution, and traffic noise): The cooling effect of blue spaces can absorb redundant heat [14]; remove pollutants, such as fine particulate matter, from the air [15]; and improve the soundscape, with road traffic, in urban spaces [16]. (2) Instoration (e.g., promotion of positive outcomes such as improved social contact or considerable physical activity): Nearby blue spaces encourage residents to engage in outdoor physical activity such as walking and jogging, which can stimulate health by producing natural feel-good hormones such as endorphins and encephalin. Blue spaces can also provide safe, attractive, and accessible places for social communication. Residents in a collective and cohesive atmosphere are likely to gain benefits such as social support and collective efficacy, which can generate positive health outcomes [17,18]. (3) Restoration (e.g., recovery from depleted attentional capacity or stress [19,20]): In environmental psychology, stress reduction theory (SRT) and attention restoration theory (ART) highlight the role of the natural environment in reducing mental stress and improving attention. SRT indicates that blue spaces in the natural environment can induce emotional experiences, thereby reducing individuals’ stress response [21]. Water was found to have restorative effects, such as being relaxing and refreshing. According to ART, four components of the blue space environment account for its restorative value, namely, being away, fascination, extent, and compatibility. ART suggests that blue spaces and other natural environments can attract and hold individuals’ attention, thereby facilitating the inhibitory mechanism in the brain and reducing attention fatigue symptoms [22].

Mounting evidence shows that blue spaces are associated with a high level of health outcomes in Western countries [12,23,24,25]. However, far less attention was paid to the relationship between blue space and the health outcomes of elderly vulnerable groups in developing and middle-income countries, especially in Asian countries. Researchers only recently investigated the linkage between blue spaces and different health outcomes (e.g., self-reported health [SRH] and mental health) in large Chinese cities. In Hong Kong, Garrett et al. (2019) found that the general health and wellbeing of elderly adults with a sea view are significantly high, but no association with mental health was observed, and no discussions on biopsychosocial pathways were provided [8]. In Shanghai, Zhang et al. (2015) observed river proximity to be inversely related to being overweight among adults aged 46–80 years, though the authors considered only the indicator of blue space accessibility [26]. In Guangzhou, Chen and Yuan (2020) determined that blue spaces are significantly associated with elderly individuals’ mental health, mediated by environmental harm reduction, stress reduction, and social contact facilitation [27]. However, the authors conducted no stratified analyses to assess whether the blueness and health relationship is modified by factors such as gender, age, and education.

The elderly’s opportunities, motivations, and ability to use blue spaces may differ between country contexts, cities, and groups, which may lead to different associations and pathways between blue spaces and the elderly’s health. Moreover, previous results on the elderly’s SRH and mental health were heterogeneous owing to influencing factors and pathways. Therefore, identifying the dose–effect relationship between blue spaces and the elderly’s SRH may not be worthwhile in the Chinese aging and high-density population context but may provide effective policy intervention implications to policymakers to maintain and enhance active aging.

In light of the research gaps, this study investigates biopsychosocial pathways and group differences linking neighborhood blue spaces and the elderly’s health using questionnaire data from a sample of 966 elderly individuals in Guangzhou, remote sensing images, a land-use map, and environmental data. This study further extends previous works in several aspects. Theoretically, this study focuses on the extent to which factors such as pollution, stress, physical activity, and social contact mediate the association between neighborhood blue spaces and the SRH of elderly individuals in the Chinese context. This study also determines whether the association is moderated by demographic characteristics (age and gender) and socioeconomic status (income). Pragmatically, this study can provide policy interventions to urban planners and landscape architects to enable elderly individuals to come in contact with blue spaces in the Chinese aging and high-density population context.

## 2. Study Design

### 2.1. Conceptual Framework

**Figure 1:**
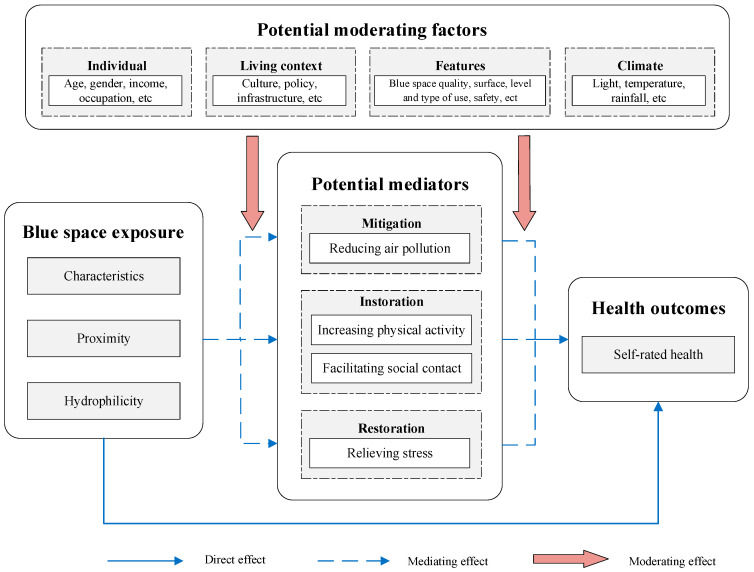
Conceptual framework.

Based on the literature review and existing studies [12,13], we created a bespoke model of neighborhood blue space and the elderly’s SRH. In the conceptual framework, we hypothesized three mediating pathways linking blue space and the elderly’s SRH, including mitigation (reducing air pollution), instoration (increasing physical activity and facilitating social contact), and restoration (reducing stress). Following Garrett et al. (2019) [8], we deconstructed blue space into three types: characteristics, proximity, and hydrophilicity. We also incorporated the four sets of effect modifiers proposed by Lachowycz et al. (2013) [28] and Hartig et al. (2014) [29], that is, individual modifiers (e.g., demographic characteristics such as age, gender, and monthly income), living context modifiers (e.g., culture, policy, and so on), blue space features (e.g., quality, safety, and so on), and climate modifiers (e.g., light, temperature, and so on). Figure 1 summarizes our conceptual framework.

### 2.2. Study Population

Guangzhou is experiencing rapid urbanization (with an urbanization rate of 86.19% in 2020) and population aging (with a proportion of the population aged 65 years or above of 13.01% in 2020). We obtained the main data used in this research from a face-to-face survey conducted in selected neighborhoods in Guangzhou, China, between November 2018 and April 2019 (including central districts such as Liwan, Yuexiu, and Haizhu; transitional districts such as Tianhe, Baiyun, and Huangpu; and marginal districts such as Panyu and Huadu). All the interviewees were between the ages of 60 and 90 years, had resided in Guangzhou for over six months, and could understand and complete the questionnaire independently. Based on a factorial ecological analysis and the criterion of the aforementioned elderly population reaching 10% using the sixth national Chinese population census, we selected 20 residential neighborhoods (shequ in Chinese) including all house types (i.e., historical neighborhoods, danwei neighborhoods, urban villages, commercial housing areas, affordable housing areas, and rural villages), as shown in Table 1.

We employed the sampling technique of multistage stratified probability proportionate to the population size to select the elderly participants in each neighborhood. In the actual investigation, we randomly selected the sample households from each sampled neighborhood using a systematic sampling approach. Next, we used the Kish grid method to choose a respondent from each sampled household. A trained interviewer administered the questionnaire in a face-to-face interview to an elderly participant. Finally, we enrolled 1000 study participants and collected a total of 966 valid questionnaires.

### 2.3. Measures

#### 2.3.1. Outcome

We measured SRH by referring to the SF-36 health survey (Medical Outcomes Study-Item Short Form Health Survey) [30]; and using a Likert scale to transform SRH into a continuous variable. The SF-36 health survey is one of the most widely used tools to assess health outcomes. The SRH assessment consisted of four dimensions: physical functioning (PF), role physical (RP), bodily pain (BP), and general health (GH). We calculated the average score of the four dimensions to assess SRH. The SF-36 questionnaire demonstrated satisfactory validity and reliability in previous studies [31]. In this study, the Cronbach’s alpha of SRH demonstrated satisfactory internal consistency (0.857).

#### 2.3.2. Neighborhood Blue Space

Guangzhou, with a subtropical monsoon climate and abundant blue space resources (e.g., rivers, lakes, and streams), is located in Southern China and has a wide water area. Following previous studies, we assessed neighborhood blue space from three dimensions: characteristics, proximity, and hydrophilicity. The multicollinearity results showed that the variance inflation factor of all the indicators was less than 4, thereby indicating that no serious multicollinearity existed.

Characteristics: This dimension included two aspects: quantity (including the three indicators of the normalized difference water index [NDWI], proportion of the water area, and per capita water area) and quality (including the indicator of landscape fragmentation). In this study, we assessed the participants’ neighborhood blue spaces using the average of the NDWI within a 1 km circular buffer around the centroid of the respondents’ residential neighborhood. To calculate the NDWI, we used satellite images from the Operational Land Imager and Thermal Infrared Sensor on Landsat 8, with a 30 m × 30 m spatial resolution, on 7 November 2018. Our reason for selecting the remote sensing images from this date is that it is consistent with the month of the survey date. In addition, on account of the subtropical monsoon climate of Guangzhou, the hydrological characteristics during the survey period (fourth quarter and first quarter) were relatively stable. The proportion of the water area is the ratio of the water area to the buffer area within the 1 km buffer zone of the neighborhood’s boundary. The per capita water area is the ratio of the water area (water area belongs under class I in the land use status classification [GB/T2010-2007] and was coded as E1) to the buffer zone (1 km buffer zone of the neighborhood’s boundary). We assessed the quality of blue space landscapes with reference to the concept of “landscape fragmentation/isolation” in landscape ecology. Landscape fragmentation may lead to a lack of habitat ecological function [32]. Furthermore, the patch separation index was the degree of fragmentation of blue spaces within the 1 km buffer zone of the neighborhood boundary, which defined the quality characteristics of the blue spaces.

Proximity: We used proximity as an indicator to assess the nearby blue spaces. The distance to the nearest water body was the Euclidean distance between the centroid of the residential neighborhood and nearest river (second-order rivers or higher), lake, or wetland.

Hydrophilicity: Hydrophilicity accounted for the blue space activities and uses. Hydrophilicity can be used to determine whether a respondent had a view of or came in contact with a nearby body of water. According to our in-depth interviews, the elderly with poor mobility were likely to stay in nearby blue spaces for a relatively long period of time. Elderly residents around hydrophilic areas can engage in hydrophilic activities, such as touching the water, walking, and talking with neighbors. Hydrophilic areas mean that the blue spaces are publicly accessible. The European Commission recommends open public spaces to be within 300 m of a residence. In our field research, we found few roadside obstacles and many paths leading to parks or rivers within 500 m around the neighborhood, so we considered using the Euclidean distance to express. Moreover, considering the investigation and neighborhood life circle in Guangzhou, we adopted 500 m as the standard for deeming a water body accessible. The hydrophilicity index was a binary variable, where 0 represented a non-hydrophilic area, and 1 represented a hydrophilic area.

#### 2.3.3. Potential Mediators

We obtained four potential mediators (air quality, stress, physical activity duration, and social contact) from the subjective and objective data, as suggested in the literature, and treated them as continuous variables in this study. First, we assessed the pollution data (air quality) in each neighborhood using the air quality index obtained from annual data comprising 62 national monitoring points in Guangzhou in 2018 and calculated the information via spatial interpolation. In this study, because spatial interpolation made an estimate within the range of the known data, we used the inverse distance weighted (IDW) method, which has the advantages of having a simple algorithm, being able to overcome estimation bias, and being easy to implement, for spatial interpolation to obtain an accurate estimation [33]. Second, we measured stress using the questions “How often did you experience emotional problems (such as feeling depressed or anxious) in the past month that affected your work and daily activities?” and “Can you concentrate when you engage in certain tasks?” We asked the participants to respond to the questions on a five-point scale (1 = never, 2 = seldom, 3 = sometimes, 4 = often, 5 = always). Third, the respondents’ physical activity duration was represented by their daily physical exercise (including walking) time in hours. Fourth, we measured social contact using the following five items rated on a five-point scale (the weight of each question for social contact was the same): “Do you have a large number of new friends?”, “Do you participate in many activities?”, “Do you know many individuals in the neighborhood?”, “Do you want to receive help from the neighborhood?”, and “Is the neighborhood cohesive?” The Cronbach’s alpha of the items was 0.792. We determined the respondents’ mean score for the social contact mediator based on the five items.

#### 2.3.4. Covariates

We incorporated a series of individual-level variables based on previous studies and our theoretical framework. We controlled for socioeconomic and demographic covariates, including age, gender, educational attainment, marital status, hukou status, monthly household income, and employment information. The summary statistics of all the variables are shown in Table 1.

### 2.4. Methods

#### 2.4.1. Statistical Analysis

Multilevel linear model: Multilevel models are specifically geared toward the analysis of data with a hierarchical structure, which can accurately distinguish and calculate the contribution of different geographical levels of elements. Considering the data nesting, in which individuals are nested in neighborhoods, we fitted a multilevel linear model using a random intercept model to assess the association between neighborhood blue spaces (neighborhood level) and the elderly’s SRH (individual level), which was necessary owing to the hierarchical structure of the data [34].

Multistep mediation analysis: To determine whether the mediators could partially or fully explain the association between neighborhood blue space and an elderly individual’s SRH, we conducted multistep mediation analysis to decompose the effect of neighborhood blue space on SRH into a direct and mediating component as well as a total effect [35]. The mediation analysis decomposed the effect of neighborhood blue space on SRH into a direct and an indirect (mediation) component. Mediation analysis can explore and evaluate biopsychosocial mechanisms, thereby elucidating potential pathways and/or aiding in policymaking.

In this study, we used Stata 14.0 (StataCorp, College Station, TX, USA) to conduct all the data analyses, which involved several steps. First, to identify the direct association, we regressed SRH (a continuous variable) on neighborhood blue space (continuous variable and binary variable; Model 1) and assessed the model performance using the Akaike information criterion. Second, we regressed each of the four mediators on the neighborhood blue space indicators and covariates (Models 2a–2d) to identify the effect of the mediators on the independent variable. Third, to identify the mediating effect, we regressed SRH outcomes on the four mediators and neighborhood blue space (Models 2a’–2d’). Fourth, to test the statistical significance of the mediating effect, we conducted bootstrap tests. The test statistic assessed whether including a mediator in the regression with neighborhood blue space reduced the health effect while the mediator remained significantly correlated. Fifth, we conducted stratified analyses to further explore the heterogeneous effect of socioeconomic status on the association between neighborhood blue space and SRH using multilevel and mediation models.

#### 2.4.2. Sensitivity Analysis

To deal with the selection bias due to the observables and check whether the relationship was robust, we used linear regressions, along with propensity score matching (PSM) [36], for the sensitivity analysis of the relationship between neighborhood blue space and the elderly’s SRH. The basic PSM process is as follows: according to the observable control variables, the probability of each sample being included in the treatment group is estimated through the logit model to obtain its tendency score. Then, the samples with the closest tendency value but belonging to two groups are matched one by one. Our decision to choose residences with blue spaces was affected by confounding variables such as individual socioeconomic attributes (e.g., gender, monthly income, age, and household registration). The elderly’s SRH may be affected by not only the built environment but also the aforementioned confounding variables. Based on urban ecosystem theory, we controlled for individual socioeconomic attributes as the confounding variables. We estimated the effect of a treatment by accounting for the covariates that predicted receiving the treatment. We defined the elderly individuals among the top 50% of those with neighborhood blue space as the treatment group and the rest as the control group. We used three matching methods (k-nearest neighbor, radius matching, and kernel matching) to match the treatment group with the control group. Finally, we obtained the average treatment effect on the treated (ATT) value of each indicator and significance.

## 3. Results

### 3.1. Characteristics of the Participants

All the characteristics variables are shown in Table 2. The 966 participants had a mean age of 69 years, 56.83% were female, and 41.41% had an educational level of “primary school or lower”. The marriage rate was 77.02%, and the retirement rate was 70.81%. The average monthly income was 2243.913 yuan, and 68.94% of the participants were local residents. For the health indicator, the average SRH score was 68.713 (SD 2.956). For the blue space indicators, neighborhood blue space included water coverage (NDWI mean = 0.747, SD ± 0.495), proportion of the water area (mean = 0.323, SD ± 0.305), per capita water area (mean = 4331.310 m^2^, SD ± 9592.703), dispersed water patch (patch separation index mean = 3.625, SD ± 2.348), distance to a river (mean = 739.9 m, SD ± 926.120), and hydrophilicity (mean = 0.550, SD ± 0.510). The respondents reported relatively low stress (mean = 4.144, SD ± 1.711), average physical activity duration (mean = 0.513 h, SD ± 1.429), and a neutral social interaction score (mean = 14.198, SD ± 2.316).

### 3.2. Multilevel Mediation Modeling

#### 3.2.1. Association between Neighborhood Blue Space and the Elderly’s SRH

With a neighborhood-level intraclass correlation of 0.1203 in the null model (without any variables), the application of the multilevel model was justifiable, meaning that neighborhood blue space indicators effectively explained the heterogeneity of the elderly’s SRH at the neighborhood level.

Model 1 shows the results of the total effect of neighborhood blue space on SRH (Table 3). In terms of the individual characteristics, gender (β = −0.285, *p* < 0.05), age (β = −0.022, *p* < 0.05), and monthly income (β = 0.734, *p* < 0.01) were significantly related to SRH outcomes. As for the neighborhood characteristics, the NDWI (β = −1.162, *p* < 0.1) was negatively associated with the elderly’s SRH, and the patch separation index (β = 0.588, *p* < 0.1) and hydrophilicity index (β = 0.140, *p* < 0.1) were positively associated with the elderly’s SRH.

#### 3.2.2. Associations between Neighborhood Blue Space and Four Mediators

Table 3 shows the results of the regression of neighborhood blue space on the four mediators. All the mediators were defined as dependent variables to verify the relationship between neighborhood blue space and the mediators (Models 2a–2d). In Model 2b, the hydrophilicity index (β = −0.314, *p* < 0.1) was negatively related to stress. In Model 2d, the patch separation index (β = 2.021, *p* < 0.05) was positively linked with social contact, whereas the hydrophilicity index (β = −0.861, *p* < 0.1) was negatively related to social contact.

#### 3.2.3. Associations between Neighborhood Blue Space, Mediators, and the Elderly’s SRH

We further examined whether the four mediators had a mediating effect (Models 2a’–2d’). Table 4 displays the results concerning whether the neighborhood blue space–SRH relationship was mediated. After we considered the mediators, we determined that neighborhood blue space remained significantly related to SRH. In Model 2a’, the NDWI (β = −1.511, *p* < 0.1) and per capita water area (β = −0.620, *p* < 0.1) were negatively related to the elderly’s SRH, whereas pollution (β = −0.080, *p* < 0.1) was negatively related to SRH. In Model 2b’, the NDWI (β = −1.039, *p* < 0.1) was negatively related to SRH, whereas stress (β = −0.464, *p* < 0.01) was negatively related to SRH. In Model 2c’, the NDWI (β = −1.136, *p* < 0.1) and per capita water area (β = −0.641, *p* < 0.1) were negatively related to the elderly’s SRH, whereas physical activity duration (β = 0.080, *p* < 0.1) was positively related to SRH. In Model 2d’, the NDWI (β = −1.216, *p* < 0.1) was negatively related to SRH, whereas the mediating effect of social contact was not significant. The results of the bootstrap tests showed that only stress was a significant mediator in the effect of the NDWI (95% confidence interval [CI] = [−0.938, −0.244]), thereby indicating that stress had a significant partial mediating effect.

### 3.3. Sensitivity Analyses

We further employed PSM to control for the selection bias in Model 1 owing to the observed characteristics. The value of the NDWI ATT was significant in the three methods of k-nearest neighbor matching (NDWI ATT = 0.400, *p* < 0.1), radius matching (NDWI ATT = 1.139, *p* < 0.05), and kernel matching (NDWI ATT = 1.098, *p* < 0.05). Regardless of the employed matching method, the effect of neighborhood blue space on the elderly’s SRH remained robust after we partially controlled for the selection bias.

### 3.4. Stratified Analysis

We stratified the analysis of the association between the elderly’s SRH and neighborhood blue space and observed statistically significant associations in the main analyses. Neighborhood blue space was associated with SRH in the age, gender, and income groups (Table 5) (Considering the number of samples and the per capita monthly income in Guangzhou, we selected the elderly whose personal monthly income was less than 3000 yuan as the low-income group, and the rest were the middle-high-income group).

In the age-stratified analysis (Table 6), no neighborhood blue space indicator was associated with SRH outcomes among the participants between the ages of 60 and 75 years, whereas distance to the nearest water body (β = −4.376, *p* < 0.05) and the hydrophilicity index (β = 1.524, *p* < 0.1) were associated with SRH outcomes only among the participants older than 75 years. In the gender-stratified analysis (Table 7), no neighborhood blue space indicator was associated with SRH outcomes in the group of male participants, whereas the patch separation index (β = 1.704, *p* < 0.05) was positively associated with SRH outcomes only in the group of female participants. In the monthly income-stratified analysis (Table 8)., per capita water area was negatively associated with SRH outcomes in the low-income group (β = −0.497, *p* < 0.1) and middle–high-income group (β = −0.930, *p* < 0.1).

As for the mediating effect presented in Table 6, Table 7 and Table 8, in the age-stratified analysis, the stress mediator could fully explain the association between neighborhood blue space and SRH outcomes among the participants older than 75 years. The bootstrap tests also confirmed this result (nearest body of water 95% CI [0.325, 0.569]; hydrophilicity index 95% CI [−0.410, −0.101]). In the gender-stratified analysis, no mediators could explain the association between neighborhood blue space and SRH outcomes in both gender groups. In the monthly income-stratified analysis, the stress mediator could fully explain the association between neighborhood blue space and SRH outcomes in both income groups. The bootstrap tests also confirmed the above results (per capita water area 95% CI [−0.674, −0.079] in the low-income group and per capita water area 95% CI [−0.552, −0.063] in the middle–high-income group).

## 4. Discussion

### 4.1. Blue Space, the Elderly’s SRH, and Mediation Role

A small but growing body of evidence demonstrates the potential health effect of engaging with blue spaces in the elderly’s later life. Our results are in line with previous studies that suggested a significant association between neighborhood blue space and the elderly’s general health [7,9,10,27,37,38], though such a relationship is not consistently observed [15]. However, evidence from our study on the blue space–health outcome association in elderly adults was scant and showed inconsistent findings. In addition, the results on the elderly’s health were heterogeneous. Some previous studies showed positive or no association between blue space and the elderly’s health. For example, a study in Scotland found that high neighborhood freshwater coverage is associated with low antidepressant prevalence [7]. Meanwhile, a study in Hong Kong determined that a view of blue spaces from home is related to satisfactory SRH among elderly adults [8]. However, a study in Spain failed to observe a significant relationship between access to blue spaces and common mental disorders or antidepressant usage among middle-aged to elderly adults [15].

Our study suggested that the blue space characteristics (NDWI) were negatively associated with the elderly’s SRH. According to certain research frameworks from previous studies and in-depth field interviews, our research results can be potentially explained by the water quality of the Pearl River in Guangzhou (blue space features modifier). We used the water quality index (WQI) to evaluate the water quality and its impact on human health, and a low WQI value poses a potential threat to human health [39]. In line with the quarterly report “Water Quality Automatic Monitoring Data” from 2016 to 2020, issued by the Department of Ecology and Environment of Guangdong Province, the water quality of the section of the Pearl River (including the Baiyun section of Liuxi River, Shijing River, and Huadi River) in Guangzhou remained stable in Class III and Class IV, with Class Inferior V (the worst class) appearing occasionally. The heavily polluted sections of the river (Classes IV, V, and Inferior V) contain dissolved oxygen and phosphorus as the major pollutants. According to our semi-structured and in-depth field interviews, some of the local elderly respondents reported that the overall river environment was unclean and worrisome, though the water quality improved in recent years. In addition, the NDWI indirectly reflected long-term exposure to the neighborhood effect. Mobility reduction and lifestyle changes in old age can increase the time spent in the neighborhood and result in considerable reliance on neighborhood resources. Compared with youths, elderly individuals with poor mobility are more likely to be exposed to poor water quality areas longer, thereby resulting in a potentially longer effect on their SRH.

Consistent with previous evidence obtained for adults, some of the functions of neighborhood blue space, such as stress relief [40], were related to the elderly’s health. For instance, a study in Germany showed that blue spaces (promenades on the river) provide restoration from daily stress [41]. Another study revealed that water scenes are associated with high perceived restorativeness of such scenes [42]. A study indicated that stress is a mediator in the effect of contact with natural outdoor environments (including blue spaces) on mental health [43]. Another study in the United States indicated that accessible blue spaces may offer restorative benefits, and visiting frequency of freshwater blue spaces is related to perceived stress [7,20]. In terms of the meditating effect pathway in our study, we observed that stress reduction played a partial mediating role in the relationship between neighborhood blue space and SRH. A poor water environment cannot effectively reduce adrenaline secretion and sympathetic nerve excitability, so it had an opposite effect on reducing stress. According to our in-depth interviews, some of the respondents reported that they did not relax too long along the riverside, which explained why the NDWI negatively affected the elderly’s SRH by relieving stress.

### 4.2. Stratified Analyses

To the best of our knowledge, the elderly’s SRH was patterned by socioeconomic and other demographic factors. In this study, we found that the relationship between neighborhood blue space and SRH varied significantly by age and income. We observed that the neighborhood blue space–SRH association was significant in the older group. These results are inconsistent with those of previous work that determined that the older elderly group benefits more from neighborhood blue spaces than the younger elderly group. For example, a study showed that high freshwater coverage in the wide neighborhood is associated with low antidepressant prevalence only among 50–64 year olds, with no significant association observed for the >65 year olds [7]. Elderly Chinese individuals with poor mobility are likely to be sensitive to distance and tend to use nearby, rather than remote, blue spaces. In addition, different indicators may lead to different association results in various groups. Perhaps owing to the minimal difference in gender perceptions of blue space safety, we did not observe any association between the gender groups. This result is also inconsistent with that of the previous works. For example, a study in Spain based on extensive visitor observations reported that more than twice as many men compared to women used an urban river pathway [44]. We observed a significant association between neighborhood blue space and SRH in both income-stratified groups. A possible explanation for this observation is that compared to the middle-high-income elderly group, the deprived low-income elderly group is in a disadvantaged position in terms of resource allocation owing to its limited ability to obtain resources, and securing improved neighborhood environmental resources is challenging. Thus, the living patterns and activities of this group depend considerably on the residential neighborhood’s natural environment. Similarly, a study in England indicated that household income is a potentially important moderator in the linkage between coastal proximity and self-reported mental health [37].

As for the mediating effect, we observed some indications that neighborhood blue space, SRH status, and stress, as a mediator, may be highly relevant to the older and low-income groups. This result is partially consistent with that of a New Zealand study on significant associations between personal income and psychological distress [45]. A possible explanation for this finding is that owing to the poor mobility and limited ability to obtain natural resources in a residential neighborhood, older and low-income elderly adults are able and willing to come in contact with the natural spaces near their place of residence to relieve stress.

### 4.3. Strengths and Limitations

Our research has three particular strengths. (1) In this study, we paid special attention to the health of the elderly. Based on the neighborhood effect, our study extended the findings of previous studies on the association between neighborhood blue spaces and the elderly’s health. (2) In the Chinese context of aging and dense populations, our empirical research explored multiple biopsychosocial pathways linking neighborhood blue space with the elderly’s SRH through the mediating effect of stress. With reference to the salutogenic effect, our study confirmed the “biophilia hypothesis” [46] and “health-related appropriations” [11]. (3) Considering the socioeconomic and demographic status of the elderly, our study further investigated potential age, gender, and income variations in the stratified analyses of the mechanisms underlying the elderly’s health in blue spaces.

Our research also has some limitations. First, the cross-sectional study had a limited ability in making causal inferences regarding our evaluated associations and mediation roles. Second, we used a self-reported questionnaire to obtain the subjective SRH data and mediators in the analysis. Third, we were unable to consider the participants’ frequency or duration of using blue spaces owing to the lack of data, which may have affected our assessment.

## 5. Conclusions

This study examined the relationship between neighborhood blue spaces and SRH among elderly individuals in Guangzhou using a questionnaire and other data in the Chinese megacity context. Our results showed that the elderly’s SRH was associated with individual- and neighborhood-level factors. Among the individual-level factors, the indicators of age, gender, and monthly income were significantly associated with the elderly’s SRH. At the neighborhood level, the characteristics of blue spaces were significantly associated with the elderly’s SRH. The multilevel mediation model showed that multiple biopsychosocial pathways existed between neighborhood blue spaces and the elderly’s SRH. Specifically, the blue space characteristics were related to the elderly’s SRH through the mediating effect of stress. The stratified analyses identified potentially susceptible groups owing to their demographic characteristics and socioeconomic status, that is, the strong association between neighborhood blue spaces and SRH outcomes in the older and low-income groups. We also observed the full mediating effect of stress in the age and income groups.

However, no evidence supported the mediating role of pollution, physical activity duration, and social contact. Hence, further research is necessary to address the remaining questions and limitations of our study, including influencing indicators and underlying mechanisms, such as the mediating effect of water pollution. Furthermore, objective data, such as health or environmental data, can be collected for the analysis, such as water samples around the neighborhood to be tested by a testing agency.

## Figures and Tables

**Table 1:** Summary statistics for the sampled neighborhoods.

Neighborhood Name	District	Jiedao	House Type	Number of Questionnaires Completed	Numbers of Aged 65 and above	Sampling Rate
Dashan village	Panyu	Dashi	Urban village	56	384	14.58%
Dengtang village	Baiyun	Zhongluotan	Rural village	52	936	5.56%
Fanghehuayuan	Liwan	Dongjiao	Affordable housing	22	454	4.85%
Guang’ao	Panyu	Luopu	Commercial housing	23	350	6.57%
Guangchuanheyuan	Liwan	Baihedong	Danwei	110	1149	9.57%
Hengsha	Huangpu	Dasha	Urban village	32	492	6.50%
Huafu	Liwan	Longjin	Historical	10	358	2.79%
Huagong	Tianhe	Wushan	Danwei	94	2636	3.57%
Huangpuhuayuan	Huangpu	Huangpu	Commercial housing	32	288	11.11%
Jiang village	Baiyun	Jianggao	Rural village	20	637	3.14%
Jinshazhou	Baiyun	Jinsha	Affordable housing	92	968	9.50%
Meilinhaian	Tianhe	Yuancun	Commercial housing	36	249	14.46%
Shanxia village	Huadu	Huadong	Rural village	49	353	13.88%
Tangdehuayuan	Tianhe	Tangxia	Affordable housing	8	232	3.45%
Tangyong village	Tianhe	Xinshi	Urban village	38	228	16.67%
Xingxian	Liwan	Hualin	Historical	29	417	6.95%
Yangrendong	Liwan	Lingnan	Historical	28	421	6.65%
Zhibei	Haizhu	Nanshitou	Danwei	128	1236	10.36%
Zhu’er village	Baiyun	Zhongluotan	Rural village	35	534	6.55%
Zhujiang	Yuexiu	Zhuguang	Historical	72	531	13.56%

**Table 2:** Summary statistics for all variables.

Measures	Variables	Mean/Proportion (Standard Deviation)	Measures	Variables	Mean/Proportion (Standard Deviation)
Covariates	Age	69.335 (7.770)	Covariates	Employment information	
	Gender			Full-time	3.62%
	Male	43.17%		Part-time	2.17%
	Female	56.83%		Retired	70.81%
	Educational Attainment			Unemployed	3.21%
	Primary school or below	41.41%		Farming	20.19%
	Junior high school	27.85%			
	Senior high school or technical secondary school	23.91%	Outcome	Self-rated health	68.713 (2.956)
	College	4.14%			
	Undergraduate university	2.59%	Predictors	NDWI	0.747 (0.495)
	Graduate and above	0.1%		Proportion of water area	0.323 (0.305)
	Marital status			Per capita water area	4331.310 m^2^ (9592.703)
	Married	77.02%		Patch separation index	3.625 (2.348)
	Single	1.24%		Distance to the nearest water body	739.900 m (926.120)
	Divorced	1.35%		Hydrophilicity index	0.550 (0.510)
	Widowed	20.39%			
	Hukou status		Mediators	Pollution	64.584 (18.683)
	Local	68.94%		Stress	4.144 (1.711)
	Non-local	31.06%		Physical activity duration	1.513 (1.429)
	Monthly household income	2243.913 (2454.823)		Social contact	14.198 (2.316)

**Table 3:** Associations between neighborhood blue space and elderly’s SRH (possible mediators).

	Model 1 DV: SRH		Model 2a DV: Pollution		Model 2b DV: Stress		Model 2c DV: Physical Activity Duration		Model 2d DV: Social Contact	
	Coef.	S.E.	Coef.	S.E.	Coef.	S.E.	Coef.	S.E.	Coef.	S.E.
Neighborhood blue space										
NDWI	−1.162 *	0.636	0.906	0.854	0.150	0.547	−0.292	0.668	1.356	1.375
Proportion of water area	0.084	0.251	0.254	0.361	−0.242	0.255	0.048	0.329	0.093	0.679
Per capita water area	−0.636	0.341	0.388	0.494	0.885 ***	0.317	0.015	0.449	−0.732	0.938
Patch separation index	0.588 *	0.439	0.062	0.845	0.055	0.370	−0.387	0.468	2.021 **	0.967
Distance to the nearest water body	0.197	0.334	−0.195	0.512	0.223	0.307	−0.113	0.436	−0.564	0.913
Hydrophilicity index	0.140 *	0.176	0.135	0.254	−0.314 *	0.182	0.235	0.223	−0.861 *	0.459
SES										
Gender (ref: male)	−0.285 **	0.137	−0.409 ***	0.127	−0.114	0.116	0.002	0.097	0.486 ***	0.186
Age	−0.022 **	0.010	−0.015 *	0.009	−0.011	0.008	−0.023 ***	0.007	0.035 ***	0.013
Hukou (ref: local)	−0.147	0.154	−0.193	0.151	0.166	0.129	0.194	0.116	−0.796 ***	0.223
Monthly household income	0.734 ***	0.282	−0.031	0.057	−0.207 ***	0.052	−0.022	0.044	0.175 **	0.084
Education (ref: primary school)										
Junior high school	−0.020	0.172	0.299 *	0.158	−0.197	0.145	0.081	0.120	−0.398 *	0.231
Senior high school or technical Secondary school	0.074	0.193	0.363 **	0.178	−0.040	0.163	0.264 *	0.135	−0.425	0.259
College	0.189	0.346	−0.565	0.421	−0.464	0.368	0.649	0.305	−0.054	0.585
Undergraduate university	0.176	0.434	0.323	0.491	0.623	0.132	0.536	0.433	0.638	0.620
Graduate and above	0.566	1.985	0.526	0.326	0.602	1.598	0.935	1.036	0.602	1.620
Marital status (ref: married)										
Single	−0.326	0.582	0.364	0.532	−0.050	0.493	−0.292	0.408	−0.013	0.782
Divorced	0.179	0.554	−0.486	0.484	−0.867	0.471	−0.128	0.391	0.370	0.242
Widowed	0.214	0.180	0.388	0.362	−0.832	0.366	−0.185	0.398	0.642	0.500
Employment information (ref: retired)										
Full-time	0.183	0.547	−0.840	0.546	0.058	0.464	−0.716 *	0.384	0.733	0.735
Part-time	−0.937 ***	0.350	0.802	0.231	0.566	0.326	0.535	0.635	0.402	0.321
Unemployed	−0.622	0.498	0.153	0.477	−0.005	0.421	−0.057	0.350	−0.636	0.670
Farming	−1.302 ***	0.380	−0.419	0.386	0.103	0.318	−0.379	0.266	−0.288	0.510
Intra-class variance	0.865	0.931	0.296	0.040	0.282	0.158	0.167	0.049	0.719	0.164
Interclass variance	3.817	0.174	2.648	0.135	2.747	0.125	1.868	0.087	6.848	0.315
Log likelihood	−2013.418		−1528.630		−1854.901		−1678.154		−2305.973	
AIC	4084.837		3108.873		3756.581		3403.437		4667.240	

Notes: Coef. = coefficient; S.E. = standard error; DV = dependent variable; SES = socio-economic status; * *p* < 0.10, ** *p* < 0.05, *** *p* < 0.01.

**Table 4:** Results of regressing neighborhood blue space on the mediators.

	Model 2a’ MV: Pollution		Model 2b’ MV: Stress		Model 2c’ MV: Physical Activity Duration		Model 2d’ MV: Social Contact	
	Coef.	S.E.	Coef.	S.E.	Coef.	S.E.	Coef.	S.E.
Neighborhood blue space								
NDWI	−1.511 *	0.773	−1.039 *	0.585	−1.136 *	0.636	−1.216 *	0.637
Proportion of water area	0.103	0.277	0.034	0.231	0.104	0.251	0.068	0.251
Per capita water area	−0.620 *	0.376	−0.271	0.315	−0.641 *	0.341	−0.621	0.341
Patch separation index	1.170	0.724	0.627	0.403	0.623	0.439	0.503	0.442
Distance to the nearest water body	0.422	0.370	0.188	0.308	0.150	0.335	0.292	0.339
Hydrophilicity index	0.153	0.195	0.028	0.162	0.128	0.176	0.184	0.178
Mediators								
Pollution	−0.080 *	0.042						
Stress			−0.464 ***	0.035				
Physical activity duration					0.080 *	0.046		
Social contact							0.038	0.024
SES								
Gender (ref: male)	−0.243	0.151	−0.335 ***	0.126	−0.286 **	0.137	−0.308 **	0.138
Age	−0.018	0.010	−0.027 ***	0.009	−0.020 **	0.010	−0.023 **	0.010
Hukou (ref: local)	−0.085	0.171	−0.070	0.141	−0.171	0.154	−0.119	0.154
Monthly household income	0.633 **	0.318	0.321	0.261	0.748 ***	0.283	0.704 **	0.283
Education (ref: primary school)								
Junior high school	0.086	0.190	−0.107	0.158	−0.026	0.172	−0.006	0.172
Senior high school or technical Secondary school	0.139	0.214	0.057	0.177	0.052	0.193	0.088	0.193
College	0.441	0.383	0.170	0.318	0.182	0.346	0.221	0.346
Undergraduate university	0.293	0.502	−0.033	0.399	0.117	0.435	0.178	0.433
Graduate and above	0.795	1.988	0.818	1.825	0.577	1.982	0.696	1.984
Marital status (ref: married)								
Single	−0.417	0.635	−0.325	0.536	−0.309	0.582	−0.319	0.582
Divorced	0.089	0.575	0.012	0.510	0.192	0.553	0.103	0.556
Widowed	0.292	0.191	0.245	0.166	0.221	0.180	0.202	0.180
Employment information (ref: retired)								
Full-time	0.055	0.650	0.223	0.503	0.238	0.547	0.154	0.547
Part-time	−0.719 *	0.432	−0.987	0.321	−0.926 ***	0.349	−0.925	0.349
Unemployed	−0.525	0.567	0.612	0.458	−0.625	0.497	−0.605	0.498
Farming	−1.051 **	0.459	−1.237	0.349	−1.284	0.380	−1.286	0.380
Intra-class variance	0.155	0.192	0.425	0.406	0.217	0.728	0.545	0.801
Interclass variance	3.774	0.189	4.226	0.147	3.804	0.173	3.807	0.173
Log likelihood	−1664.3525		−1932.3221		−2011.8911		−2012.1621	
AIC	3388.705		3924.644		4083.782		4084.324	

Notes: Coef. = coefficient; S.E. = standard error; MV = Mediating variable; SES = socio-economic status; * *p* < 0.10, ** *p* < 0.05, *** *p* < 0.01.

**Table 5:** Results of stratified multilevel models on the relationship between neighborhood blue space and SRH outcome.

Strata	Sub-Strata	NDWI	Proportion of Water Area	Per Capita Water Area	Patch Separation Index	Distance to the Nearest Water Body	Hydrophilicity Index
Age	60–75 years old	−0.755	0.752	−0.955	0.894	−0.728	0.473
	>75 years old	−0.489	2.650	−0.65	1.862	−4.376 **	1.524 *
Gender	male	−2.013	0.043	−0.561	0.626	0.261	0.388
	female	−0.335	1.012	−0.708	1.704 **	−1.535	0.544
Monthly	≤3000 yuan	−0.024	1.018	−0.497 *	2.116	−1.653	0.629
income	>3000 yuan	−1.275	0.488	−0.930 *	0.698	−0.074	0.305

Notes: * *p* < 0.10, ** *p* < 0.05.

**Table 6:** Associations between neighborhood blue space, mediators, and SRH in age groups.

	Model 3-1a and Model 3-2a DV: Pollution		Model 3-1b and Model 3-2b DV: Stress		Model 3-1c and Model 3-2c DV: Physical Activity Duration		Model 3-1d and Model 3-2d DV: Social Contact	
	Coef. (60–75 Years)	Coef. (>75 Years)	Coef. (60–75 Years)	Coef. (>75 Years)	Coef. (60–75 Years)	Coef. (>75 Years)	Coef. (60–75 Years)	Coef. (>75 Years)
Neighborhood blue space								
NDWI	−1.440	−0.083	−0.782	−2.026	0.581	−0.583	0.685	3.998 *
Proportion of water area	1.836 **	0.902	−1.020	−3.358 **	1.115	1.250	−0.014	2.200
Per capita water area	−1.877 **	−0.381	1.651 ***	0.666	−1.302 **	−0.527	−0.496	−0.109
Patch separation index	3.485 **	−1.183	0.596	−0.511	0.010	0.330	0.801	0.829
Distance to the nearest water body	−2.352 ***	0.749	0.353	3.748 **	−1.159 *	−2.684 *	−0.282	−1.725
Hydrophilicity index	0.967 ***	−0.403	−0.485	−1.323 *	0.621 **	1.554 **	−1.192 **	−1.558
	Model 3-1a’ and model 3-2a’ MV: Pollution		Model 3-1b’ and model 3-2b’ MV: Stress		Model 3-1c’ and model 3-2c’ MV: Physical Activity Duration		Model 3-1d’ and model 3-2d’ MV: Social Contact	
	Coef. (60–75 years)	Coef. (>75 years)	Coef. (60–75 years)	Coef. (>75 years)	Coef. (60–75 years)	Coef. (>75 years)	Coef. (60–75 years)	Coef. (>75 years)
Neighborhood blue space								
NDWI	−3.110	2.418	−1.077	−1.624	−0.805	−0.466	−0.792	−0.365
Proportion of water area	0.195	6.018 **	0.332	0.768	0.663	2.600	0.756	2.718
Per capita water area	−0.923	−2.525	−0.275	−0.285	−0.845	−0.638	−0.923	−0.662
Patch separation index	3.270	0.304	1.139*	1.575 *	0.890	1.849 *	0.847	1.888 *
Distance to the nearest water body	−1.014	−6.129 ***	−0.582	−2.276	−0.629	−4.269 **	−0.713	−4.430 **
Hydrophilicity index	0.505	2.371 **	0.273	0.782	0.421	1.461	0.558	1.475
Mediators								
Pollution	−0.112 **	0.097						
Stress			−0.411 ***	−0.560 ***				
Physical activity duration					0.089 *	0.040		
Social contact							0.068 **	−0.030

Notes: Coef. = coefficient; DV = dependent variable; MV = mediating variable; * *p* < 0.10, ** *p* < 0.05, *** *p* < 0.01.

**Table 7:** Associations between neighborhood blue space, mediators, and SRH in gender groups.

	Model 4-1a and Model 4-2a DV: Pollution		Model 4-1b and Model 4-2b DV: Stress		Model 4-1c and Model 4-2c DV: Physical Activity Duration		Model 4-1d and Model 4-2d DV: Social Contact	
	Coef. (Male)	Coef. (Female)	Coef. (Male)	Coef. (Female)	Coef. (Male)	Coef. (Female)	Coef. (Male)	Coef. (Female)
Neighborhood blue space								
NDWI	−1.478	−0.359	0.683	−1.665 *	−0.518	0.152	1.028	1.264
Proportion of water area	2.495 *	1.519	−1.201	−0.870	0.921	0.483	0.795	−1.022
Per capita water area	−1.703	−1.412	1.843 ***	0.619	−0.734	−1.071 *	0.214	−0.380
Patch separation index	2.621	0.827	0.158	0.197	−0.019	0.216	1.214	0.448
Distance to the nearest water body	−2.335 *	−1.227	0.913	0.194	−0.919	−0.985	−0.728	0.262
Hydrophilicity index	1.001 **	0.650	−0.771 *	−0.214	0.548	0.725 *	−0.912	−2.208 ***
	Model 4-1a’ and model 4-2a’ MV: Pollution		Model 4-1b’ and model 4-2b’ MV: Stress		Model 4-1c’ and model 4-2c’ MV: Physical Activity Duration		Model 4-1d’ and model 4-2d’ MV: Social Contact	
	Coef. (male)	Coef. (female)	Coef. (male)	Coef. (female)	Coef. (male)	Coef. (female)	Coef. (male)	Coef. (female)
Neighborhood blue space								
NDWI	−1.990	−2.028	−1.622	−1.102	−1.954	−0.358	−2.000	−0.384
Proportion of water area	0.115	1.555	−0.313	0.611	0.270	0.937	0.205	1.052
Per capita water area	−0.539	−1.550	0.164	−0.422	−0.715	−0.540	−0.710	−0.693
Patch separation index	0.831	3.8271	0.724	1.795	0.650	1.670 **	0.583	1.686 **
Distance to the nearest water body	0.266	−2.314 *	0.481	−1.445	0.032	−1.381	0.094	−1.545
Hydrophilicity index	0.421	0.836	0.076	0.445	0.450	0.430	0.488	0.629
Mediators								
Pollution	−0.052	−0.078						
Stress			−0.468 ***	−0.460 ***				
Physical activity duration					−0.023	0.156 **		
Social contact							0.055	0.038

Notes: Coef. = coefficient; DV = dependent variable; MV = mediating variable; * *p* < 0.10, ** *p* < 0.05, *** *p* < 0.01.

**Table 8:** Associations between neighborhood blue space, mediators, and SRH in income groups.

	Model 5-1a and Model 5-2a DV: Pollution		Model 5-1b and Model 5-2b DV: Stress		Model 5-1c and Model 5-2c DV: Physical Activity Duration		Model 5-1d and Model 5-2d DV: Social Contact	
	Coef. (Low Income)	Coef. (Middle-High Income)	Coef. (Low Income)	Coef. (Middle-High Income)	Coef. (Low Income)	Coef. (Middle-High Income)	Coef. (Low Income)	aCoef. (Middle-High Income)
Neighborhood blue space								
NDWI	−0.817	1.848	−1.798 *	−0.182	0.412	0.211	1.081	0.870
Proportion of water area	0.455	3.260 **	−1.331	−1.835	0.835	1.316	−1.074	−0.728
Per capita water area	0.607	2.565 ***	1.509 **	1.337 *	−1.098 **	−1.204 *	−0.172	0.726
Patch separation index	−0.719	1.034	0.761	−0.095	−0.163	0.158	0.029	0.506
Distance to the nearest water body	0.049	−3.109 **	1.618	0.388	−1.725 *	−1.382	0.854	1.006
Hydrophilicity index	0.132	1.196 **	−0.369	−0.792 *	0.492	1.159 ***	−1.841 **	−1.928 **
	Model 5-1a’ and model 5-2a’ MV: Pollution		Model 5-1b’ and model 5-2b’ MV: Stress		Model 5-1c’ and model 5-2c’ MV: Physical Activity Duration		Model 5-1d’ and model 5-2d’ MV: Social Contact	
	Coef. (low income)	Coef. (middle-high income)	Coef. (low income)	Coef. (middle-high income)	Coef. (low income)	Coef. (middle-high income)	Coef. (low income)	Coef. (middle-high income)
Neighborhood blue space								
NDWI	−1.506	−0.985	−0.784	−1.359	−0.001	−1.284	0.039	−1.342
Proportion of water area	0.352	0.492	0.528	−0.356	1.014	0.428	1.198	0.508
Per capita water area	−0.026	−0.800	−0.126	−0.314	−0.396	−0.875	−0.588	−0.954
Patch separation index	3.271	0.813	2.472 ***	0.654	2.138 ***	0.691	2.108 **	0.688
Distance to the nearest water body	−1.456	−0.148	−0.919	0.104	−1.382	−0.012	−1.719	−0.127
Hydrophilicity index	0.597	0.314	0.509	−0.059	0.597	0.253	0.744	0.415
Mediators								
Pollution	0.014	−0.116 **						
Stress			−0.477 ***	−0.460 ***				
Physical activity duration					0.179 **	0.045		
Social contact							0.031	0.058 *

Notes: Coef. = coefficient; DV = dependent variable; MV = mediating variable; * *p* < 0.10, ** *p* < 0.05, *** *p* < 0.01.

## Data Availability

Not applicable.
